# A Purpose-Synthesised Anti-Fibrotic Agent Attenuates Experimental Kidney Diseases in the Rat

**DOI:** 10.1371/journal.pone.0047160

**Published:** 2012-10-10

**Authors:** Richard E. Gilbert, Yuan Zhang, Spencer J. Williams, Steven C. Zammit, David I. Stapleton, Alison J. Cox, Henry Krum, Robyn Langham, Darren J. Kelly

**Affiliations:** 1 Keenan Research Centre, Li Ka Shing Knowledge Institute, St. Michael’s Hospital, Toronto, Ontario, Canada; 2 Department of Medicine, University of Melbourne, St. Vincent’s Hospital, Fitzroy, Victoria, Australia; 3 Bio21 Molecular Science and Biotechnology Institute, School of Chemistry, University of Melbourne, Parkville, Victoria, Australia; 4 Department of Physiology, University of Melbourne, Parkville, Victoria, Australia; 5 Centre of Cardiovascular Research and Education in Therapeutics, School of Public Health, Monash University, Melbourne, Australia; 6 Fibrotech Therapeutics Pty Ltd, Melbourne, Australia; INSERM, France

## Abstract

**Background and Purpose:**

Locally-active growth factors have been implicated in the pathogenesis of many diseases in which organ fibrosis is a characteristic feature. In the setting of chronic kidney disease (CKD), two such pro-fibrotic factors, transforming growth factor-ß (TGF-ß) and platelet-derived growth factor (PDGF) have emerged as lead potential targets for intervention. Given the incomplete organ protection afforded by blocking the actions of TGF-ß or PDGF individually, we sought to determine whether an agent that inhibited the actions of both may have broader effects in ameliorating the key structural and functional abnormalities of CKD.

**Experimental Approach:**

Accordingly, we studied the effects of a recently described, small molecule anti-fibrotic drug, 3-methoxy-4-propargyloxycinnamoyl anthranilate (FT011, Fibrotech Therapeutics, Australia), which should have these effects.

**Key Results:**

In the in vitro setting, FT011 inhibited both TGF-ß1 and PDGF-BB induced collagen production as well as PDGF-BB-mediated mesangial proliferation. Consistent with these in vitro actions, when studied in a robust model of non-diabetic kidney disease, the 5/6 nephrectomised rat, FT011 attenuated the decline in GFR, proteinuria and glomerulosclerosis (p<0.05 for all). Similarly, in the streptozotocin-diabetic Ren-2 rat, a model of advanced diabetic nephropathy, FT011 reduced albuminuria, glomerulosclerosis and tubulointerstitial fibrosis.

**Conclusions and Implications:**

Together these studies suggest that broadly antagonising growth factor actions, including those of TGF-ß1 and PDGF-BB, has the potential to protect the kidney from progressive injury in both the diabetic and non-diabetic settings.

## Introduction

Chronic kidney disease (CKD) is a major cause of morbidity, recurrent hospitalisation and accelerated death, affecting 10–11% of the population in both Europe and the United States [1]. In a substantial proportion of such patients, deteriorating kidney function leads to the development of end-stage kidney disease (ESKD), requiring dialysis or transplantation to preserve life. Studies conducted almost 20 years ago highlighted the importance of blood pressure control and blockade of the renin-angiotensin system in attenuating the progression of CKD towards its end stage. Unfortunately, while substantial progress has been made in our understanding of renal pathophysiology, there has been little in the way of new therapies since that time.

Possessing only a limited capacity for regeneration, sustained or repeated injury to the kidney leads to the deposition of excessive quantities of extracellular matrix in both the glomerulus and tubulointerstitium. These expansive pathological changes, recognised histologically as glomerulosclerosis and tubulointerstitial fibrosis, encroach on surrounding structures inevitably leading to capillary rarefaction with consequent hypoxia, tubular atrophy and inflammatory cell infiltration [2]. These structural changes, in turn, result in a loss of GFR that is frequently, though not invariably, accompanied by worsening proteinuria. This final common pathway, occurring in most forms of chronic kidney disease, ensues almost regardless of primary aetiology, developing in response to seemingly diverse disorders that include metabolic, immunological and infectious causes [2], [3].

Studies conducted over more than a decade have consistently indicated a major role for the prosclerotic growth factor, transforming growth factor-ß (TGF-ß) in renal fibrosis and dysfunction [4]. However, other locally-active growth factors have also been implicated in the fibrogenic process, particularly platelet-derived growth factor (PDGF), a potent inducer of matrix synthesis and the proliferation of fibrogenic mesenchymal cells such as fibroblasts and mesangial cells [5]. Consistent with these actions, kidney tissue from a range of human and experimental kidney diseases demonstrates increased expression in the components of both the TGF-ß [6] and PDGF pathways [7] such that each has become an important therapeutic targets in an attempt to develop new therapies for chronic kidney disease [7], [8]. It remains uncertain, however, whether it is optimal for a therapeutic agent to antagonise TGF-ß or PDGF separately or in combination.

In addition to a range of relatively specific antagonists of both the TGF-ß and PDGF systems, a number of other agents whose mechanisms of action are less well understood have also been shown to inhibit the actions of these two growth factors to varying extents. Among these compounds is tranilast, marketed in Japan by Kissei Pharmaceutical Co., Ltd. for the treatment of allergic diseases since 1982 and more recently for the treatment of keloid/hypertrophic scars also with a spectrum of action that includes the inhibition of TGF-ß and PDGF’s actions [9]. To optimize the anti-fibrotic effects of this compound, we synthesised a series of cinnamoyl anthranilate derivatives of tranilast, based initially on their ability to inhibit TGF-ß induced collagen production [10]. These studies led to the generation of several derivatives with superior potency and reduced cellular toxicity relative to tranilast [10]. In pilot studies, one of the derivatives, 3- methoxy-4-propargyloxycinnamoyl anthranilate (FT011, Fibrotech Therapeutics, Melbourne, Australia) was shown to reduce albuminuria in a rat model of diabetic nephropathy [10]. Following on from these findings, we undertook the present series of studies to firstly assess the ability of FT011 to inhibit PDGF’s actions in addition to those of TGF-ß and secondly to examine its efficacy in animal models of both diabetic and non-diabetic CKD that are characterised by fibrosis.

## Methods

### Mesangial Cells Culture

A well-characterized cloned mesangial cell line (1097) isolated from Sprague-Dawley rats [11] was used between passages 20 and 40. Cells were cultured in Dulbecco’s Modified Eagle’s Medium (DME, Invitrogen, Grand Island, NY, USA) with heat-inactivated fetal calf serum (FCS), 100 U/mL penicillin, and 100 µg/mL streptomycin in humidified 5% CO_2_ atmosphere at 37°C. Mesangial cells were plated out in 24-well plates in DME/5% FCS and allowed to adhere overnight. The sub-confluent cells were then starved overnight in DME/0.5% FCS prior to commencement of studies.

### Proliferation

To determine the effects of FT011 on mesangial cell proliferation, cells were treated with 10–100 µM of FT011 for 4 hours prior to the addition of recombinant PDGF-BB 50 ng/mL (Sigma, St Louis, MO). Cells were incubated for a further 20 hours and ^3^H-thymidine (1 µCi/well, Amersham Bioscience, Little Chalfont, Buckinghamshire, UK) was added for the last 4 hours of culture. Cells were washed twice in ice cold phosphate-buffered saline (PBS), incubated in ice cold 10% TCA for 30 minutes, followed by a final wash in ice cold 10% TCA. The cells were then dissolved in 1 M sodium hydroxide. This solution was then neutralized with hydrochloric acid, and scintillation counting was performed. Replicates of three wells were used. Cell viability was assessed by trypan blue exclusion.

### Collagen Synthesis

^3^H-proline incorporation was used as an index of collagen production. Mesangial cells were plated, cultured, and starved as for thymidine incorporation except that 150 µM L-ascorbic acid was incubated in the starve medium for 4 hours. Cells were treated with 10–100 µM FT011 for 4 hours prior to the addition of PDGF-BB (50 ng/mL) or TGF-β1 (5 ng/ml, R & D systems) and 1 µCi/well L-[ 2, 3, 4, 5-3H] proline (Amersham). Cells were cultured for a further 44 hours and washed and counted as for thymidine incorporation. Replicates of three wells were used. Proline incorporation was adjusted for protein content. An aliquot of the remaining lysate was neutralised with 1 M HCL and assayed in a BioRad (Bradford) Protein Assay. Results were expressed as cpm 3H-proline/µg of total protein.

### Animals

The animal studies were conducted with the approval from the Animal Welfare and Ethics Committee (St Vincent’s Hospital and the National Health and Medical Research Foundation of Australia). All rats received normal rat chow (Certified Rodent Diet #5002, LabDiet, USA) and drinking water *ad libitum.* Animals were housed in a stable environment maintained at 22±1°C with a 12-hour light/dark cycle commencing at 6 a.m.

### Sub-total Nephrectomy Rats

Forty male Sprague-Dawley rats weighing 200–250 g were randomised to receive sub-total nephrectomy (STNx) or sham surgery. Anaesthesia was achieved with 3% isoflurane/97% oxygen in a tidal volume of 1 ml/100 g body weight. The control group underwent sham surgery (n = 20) consisting of laparotomy and manipulation of both kidneys before wound closure. The other 20 rats underwent STNx performed by right subcapsular nephrectomy and infarction of approximately 2/3 of the left kidney by selective ligation of two out of the three to four extrarenal branches of the left renal artery [12]. Two weeks post-surgery, sham and STNx animals (n = 20) were then randomly assigned to 2 groups each (n = 10) to receive either FT011 (200 mg/kg/day, bid gavage) or vehicle (1% CMC) for 12 weeks.

### Diabetic Nephropathy Rats

Forty, 6-week old female, heterozygous (mRen-2)27 rats (St. Vincent’s Hospital Animal House, Melbourne, Australia) were assigned to receive either 55 mg/kg of streptozotocin (STZ) (Sigma, St. Louis, USA) diluted in 0.1 M citrate buffer, pH 4.5 or citrate buffer alone (non-diabetic control) by tail vein injection following an overnight fast. Within 24 hours post-STZ or citrate buffer injection, control and diabetic groups (n = 20) were further randomised to receive either treatment with FT011 (200 mg/kg/day bid gavage) or vehicle for 16 weeks. Each week, rats were weighed and their blood glucose levels were measured (Accu-check Advantage II Blood Glucose Monitor, Roche Diagnostics, USA) and only STZ-treated animals with blood glucose >15 mmol/L were considered diabetic. Every 4 weeks, systolic blood pressure (SBP) was determined in preheated conscious rats via tail-cuff plethysmography using a non-invasive blood pressure (NIBP) controller and Powerlab (AD instruments, NSW, Australia). Haemoglobin A1c (HbA1c) was measured by HPLC at the end of the study in the Department of Pathology, St Vincent’s Hospital, Melbourne, Australia. Diabetic rats received 2 to 3 times a week injection of insulin (2–4 units intraperitoneally; Humulin NPH, Eli Lilly and Co., Indianapolis, IN) to reduce mortality and to promote weight gain.

### Proteinuria/Albuminuria

STNx rats were individually housed in metabolic cages at the end of the study. In the prevention study, diabetic rats were caged at 4, 8 12 and 16 weeks. Rats were habituated for 2 to 3 hours and urine collected over 24 hours. Animals continued to have free access to tap water and standard laboratory chow during this period. After 24 hours in metabolic cages, an aliquot of urine (5 ml) was collected from the 24-hour urine sample and stored at –20°C for subsequent analysis. For STNx rats, proteinuria was measured by the Department of Pathology, St Vincent’s Hospital, Melbourne, Australia. Albuminuria excretion rate (AER) from diabetic nephropathic rats was measured by radio-immunoassay as previously performed in our group [13]. Both proteinuria and AER were expressed as mg/24 hours.

### Glomerular Filtration Rate (GFR)

Prior to animals sacrifice, GFR was determined by injecting a single shot of ^99^Tc-DTPA into the tail vein and the blood was sampled after 43 minutes as previously described [12] and expressed as ml/minute.

### Tissue Preparation

Rats were euthanized (Nembutal 60 mg/kg body wt i.p. Boehringer-Ingelheim, Australia). Kidneys were excised, decapsulated, sliced transversely, half of the kidney was snap-frozen for molecular biology and the other half was immersion fixed with 10% neutral buffered formalin (NBF) and paraffin-embedded for subsequent light microscopic evaluation.

### Histopathology

Histopathological changes in kidney were assessed in a masked protocol. Sections were stained with either Periodic Acid Schiff’s (PAS) and modified Masson’s trichrome staining to demonstrate mesangial matrix expansion and interstitial fibrosis, respectively.

### Glomerulosclerotic Index

In 4 µm kidney sections stained with PAS, 70 to 80 glomeruli from each rat were examined in a masked protocol. The extent of sclerosis in each glomerulus was subjectively graded on a scale of 0 to 4, as previously described [12] with Grade 0, normal; Grade 1, sclerotic area up to 25% (minimal); Grade 2, sclerotic area 25–50% (moderate); Grade 3, sclerotic area 50–75% (moderate to severe) and Grade 4, sclerotic area 75–100% (severe). A glomerulosclerotic index (GSI) was then calculated using the formula:
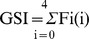
where Fi is the % of glomeruli in the rat with a given score (i).

### Immunostaining for Macrophage and Osteopontin

Four µm kidney sections were placed into histosol to remove the paraffin wax, hydrated in graded ethanol and immersed into distilled water before being incubated for 20 minutes with normal goat serum (NGS) diluted 1∶10 with 0.1 M PBS at pH 7.4. Sections were then incubated for 18 hours at 4°C with specific primary monoclonal rat macrophage marker (ED-1, 1∶300 Serotec, Raleigh NC, USA) and antibody to osteopontin (1∶2000, gift from The University of Iowa, IA, USA) as previously described [14]. The following day the sections were thoroughly washed in PBS (3×5 minutes changes), incubated with 3% hydrogen peroxide for 10 minutes to block endogenous peroxide, then rinsed with PBS (2×5 minutes), and incubated with biotinylated goat anti-mouse IgG antibody (DAKO, Carpinteria CA), diluted 1∶200 with PBS. Sections were then rinsed with PBS (2 x 5 minutes) followed by incubation with an avidin-biotin peroxidase complex (Vector Laboratories, Burlingame, CA, USA), diluted 1∶200 with PBS. Following rinsing with PBS (2 x 5 minutes), localization of the peroxidase conjugates was achieved by using DAB as a chromagen, for 1–3 minutes. Sections were rinsed in tap water for 5 minutes to stop reaction and then counterstained in Mayer’s haematoxylin, differentiated in Scott’s tap water, dehydrated, cleared and mounted in Depex. Sections incubated with 1∶10 NGS instead of the primary antiserum served as the negative control.

### Quantitation of interstitial fibrosis

In 4 µm kidney sections stained with Masson’s trichrome, 10 random non-overlapping fields from cortex (10 rats per group) were captured and digitised using a Carl Zeiss microscope attached to AxioCamMRc5 digital camera (Carl Zeiss, North Ryde, NSW, Australia) under 200×magnification. Digital images were then loaded onto a Pentium D Dell computer. An area of blue in cortex of the kidney was selected for its colour range and the proportional area of the selected colour range was then quantified using image analysis (AxioVision Release 4.8.1; Carl Zeiss, North Ryde, NSW, Australia) based on the method adapted from Lehr et al. [15], [16]. Data were expressed as percentage change per area.

### Quantitation of Interstitial Macrophage Infiltration and Tubular Osteopontin

Macrophage number in tubulointerstitium of the kidney was quantitated by counting the number of macrophages in 10 fields under light microscopy with a magnification x200 per animal from each group and expressed as numbers per field. Positive immunostaining for osteopontin in kidney sections was quantified using computer-assisted image analysis, as previously described [15]. Briefly, 10 random non-overlapping fields in the cortex of the kidney were captured, and digitised using a Carl Zeiss microscope attached to AxioCamMRc5 digital camera (Carl Zeiss, North Ryde, NSW, Australia) with 200×power. Digital images were then loaded onto a Pentium D Dell computer. An area of brown on immunostained sections was selected for its colour range and the proportional area of tissue with their respective range of colour was then quantified. Calculation of the proportional area stained brown was then determined using image analysis (AxioVision Release 4.8.1; Carl Zeiss, North Ryde, NSW, Australia) with method adapted from Lehr described previously [15], [16] and expressed as percentage area [17].

### Statistics

Data are expressed as mean ± SEM unless otherwise stated. Statistical significance was determined by one-way ANOVA with Fishers post-hoc comparison. Albuminuria was skew distributed and was analysed following log transformation and presented as geometric means x/÷ tolerance factors. Analyses were performed using Statview II + Graphics package (Abacus Concepts, Berkeley, California) on an Apple Macintosh G4 computer (Apple Computer, Inc., Cupertino, California). A p-value <0.05 was regarded as statistically significant.

## Results

### *In vitro* Studies

#### Effects of FT011 on PDGF-BB and TGF-β1 induced collagen production

**Figure 1:**
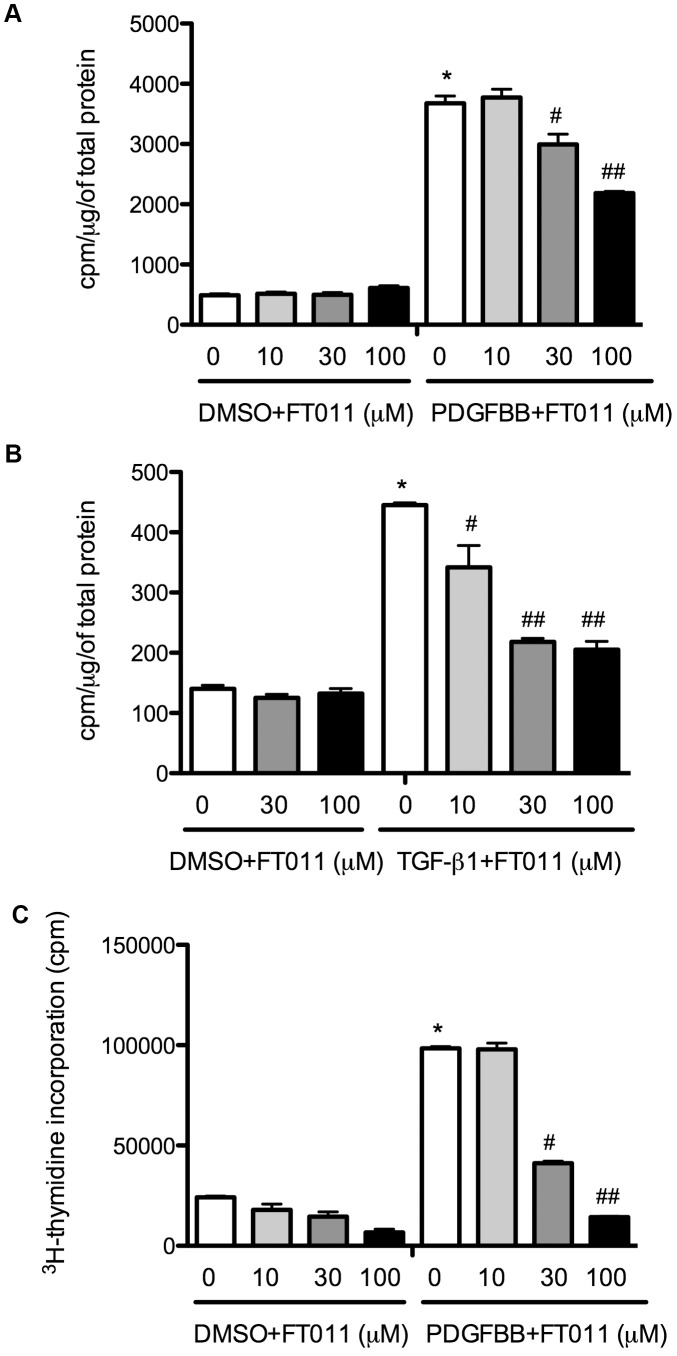
^3^H-proline and ^3^H-thymidine incorporation. PDGF BB (Figure 1A) or TGF-β1 (Figure 1B) stimulated collagen production in cultured mesangial cells with and without FT011 treatment as assessed by ^3^H-proline incorporation. Data are expressed as mean ± SEM. *p<0.001 versus DMSO with or without FT011 (10, 30 or 100 µM), **^#^** *p*<0.01 versus PDGFBB or TGF-β1 and **^##^** *P*<0.01 versus PDGFBB with FT011 (30 µM) or TGF-β1 with FT011 (10 µM). PDGF-BB stimulated DNA synthesis in cultured mesangial cells (Figure 1C) with and without FT011 treatment as assessed by ^3^H-thymidine incorporation. Data are expressed as mean ± SEM. *p<0.001 versus DMSO with or without FT011 (10, 30 or 100 µM), **^#^** *p*<0.01 versus PDGF-BB, **^##^** *P*<0.01 versus PDGFBB with FT011 (30 µM).

PDGF-BB stimulation resulted in a 7.5 -fold increase in collagen synthesis when compared to unstimulated mesangial cells, P<0.001 (Figure 1A). Similarly TGF-β1 stimulation increased collagen synthesis by 3.15 -fold, P<0.001 (Figure 1B). Pre-treatment of mesangial cells with FT011 was associated with a reduction in collagen synthesis in response to PDGF-BB or TGF-β1 stimulation in a dose dependent manner. Treatment with FT011 prior to PDGF-BB stimulation, reduced collagen synthesis by 18% at a 30 µM, and by 40% at a 100 µM when compared to PDGF-BB stimulated mesangial cells without treatment of FT011 P<0.01 (Figure 1A). Similarly, treatment with FT011 prior to TGF-β1 stimulation, reduced collagen synthesis by 23% at a 10 µM, by 50% at a 30 µM and by 54% at a 100 µM when compared to TGF-β1 stimulated mesangial cells without treatment of FT011 P<0.01 (Figure 1B).

#### Effect of FT011 on PDGF-BB stimulated cell proliferation

PDGF-BB stimulation induced a 4-fold increase in cell proliferation when compared to unstimulated mesangial cells, P<0.001 (Figure 1C). Treatment with FT011 prior to PDGF-BB stimulation, reduced cell proliferation by 58% at a 30 µM, and by 85% at a 100 µM when compared to PDGF-BB stimulated mesangial cells without treatment (P<0.01, Figure 1C).

### *In vivo* Studies

#### Animal characteristics

**Figure 2:**
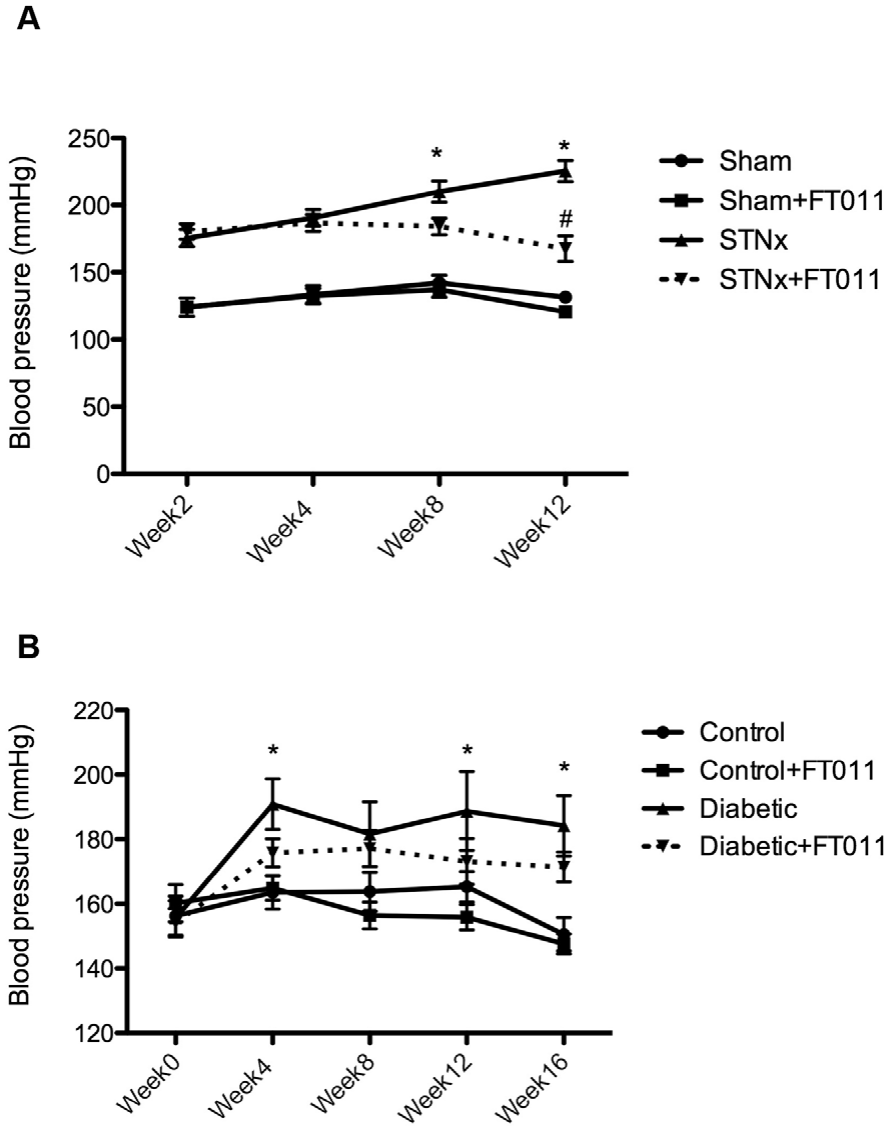
Systolic blood pressure in STNx and diabetic rats. Systolic blood pressure in STNx and diabetic rats was progressively elevated during the course of the study (Figure 2A and Figure 2B) when compared to sham and control rats, respectively. Treatment of STNx rats with FT011 prevented elevation in blood pressure (Figure 2A), while FT011 did not affect blood pressure in diabetic Ren-2 rats through the course of the study (Figure 2B). Data are expressed as mean ± SEM. *P<0.01 compared with sham or control, **^#^**P<0.05 versus untreated STNx rats.

STNx rats developed proteinuria and high blood pressure along with declining GFR (Table 1 and Figure 2A). Without affecting body weight gain, treatment of STNx rats with FT011 was associated with a significant reduction in urinary protein, attenuation of increasing blood pressure and declining GFR when compared to vehicle treated STNx rats (Table 1 and Figure 2A). Blood pressure was similar in both STNx groups 2 weeks post nephrectomy but began to diverge thereafter with lower pressures among FT011-treated animals. In sham rats and sham rats treated with FT011, no mortality was observed. In STNx rats treated vehicle 6/18 rats died during the treatment period, while 4/18 rats treated with FT011 died.

**Table 1:** Animal characteristics of subtotal nephrectomised rats.

Group	Body weight (g)	Body weight gainover 12 weeks (g)	Systolic BP (mmHg)	GFR (ml/min)	Proteinuria (mg/d)
Sham	491±11	223±9	131±4	4.83±0.29	21±1.4
Sham+FT011	485±18	228±16	120±3	4.86±0.14	24±2.2
STNx	443±31*	226±23	225±7*	0.38±0.14*	366±42*
STNx+FT011	428±15*	203±18	167±9*†	1.19±0.14*†	180±38*†

Animal characteristics in sham and STNx rats treated with and without FT011. Data are expressed as mean ± SEM. *P<0.05 versus sham rats treated with or without FT011,

† p<0.05 versus untreated STNx rats.

**Figure 3:**
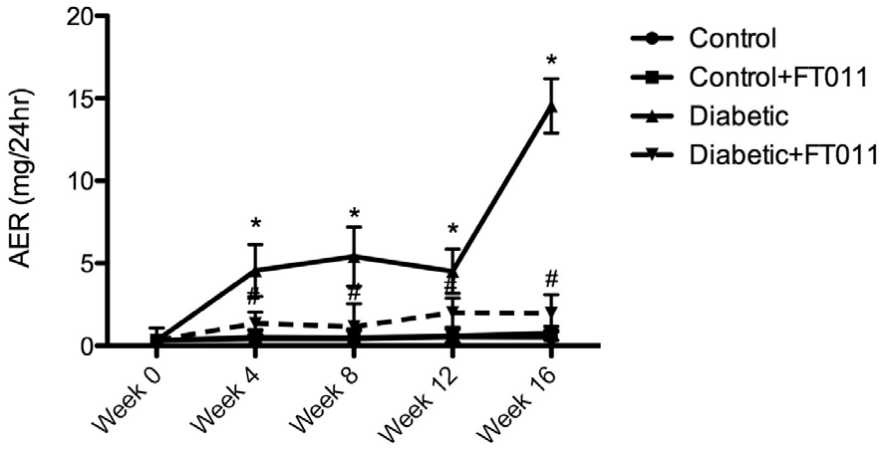
Albuminuria in diabetic rats. Albuminuria, expressed as mean ± SEM, developed in untreated diabetic animals during the course of the study and to a lesser extent in rats receiving FT011. **^#^** *P*<0.05 versus vehicle treated diabetic rats.

In comparison with control animals, diabetic rats had elevated blood pressure, plasma glucose, HbA1c, GFR and kidney weight to body weight ratio, which were all unaffected by the treatment with FT011 (Table 2 and Figure 2B). Untreated diabetic animals developed progressively worsening albuminuria during the course of the study that was attenuated by FT011 at all time points (Figure 3).

**Table 2:** Animal characteristics of diabetic Ren-2 rats.

Group	Body weight (g)	SBP (mmHg)	Plasma Glucose (mM)	HbA_1c_ (%)	GFR (ml/min)	KW/BW
Control	294±11	150±5	5±0.2	3.8±0.03	3.4±0.2	0.34±0.03
Control+FT011	309±7	147±3	7±0.2	3.2±0.04	3.7±0.1	0.39±0.03
Diabetes	281±22*	184±7*	33±0.2*	10.7±0.27*	5.6±0.3*	0.70±0.07*
Diabetic+FT011	278±12*	171±5*	30±0.5*	9.7±0.28*	5.6±0.2*	0.72±0.11*

Animal characteristic in control and diabetic Ren-2 rats treated with and without FT011. Data are expressed as mean ± SEM. *P<0.05 versus treated or untreated control rats. SBP, systolic blood pressure; KW/BW = kidney to body weight ratio.

#### Renal structure

**Figure 4:**
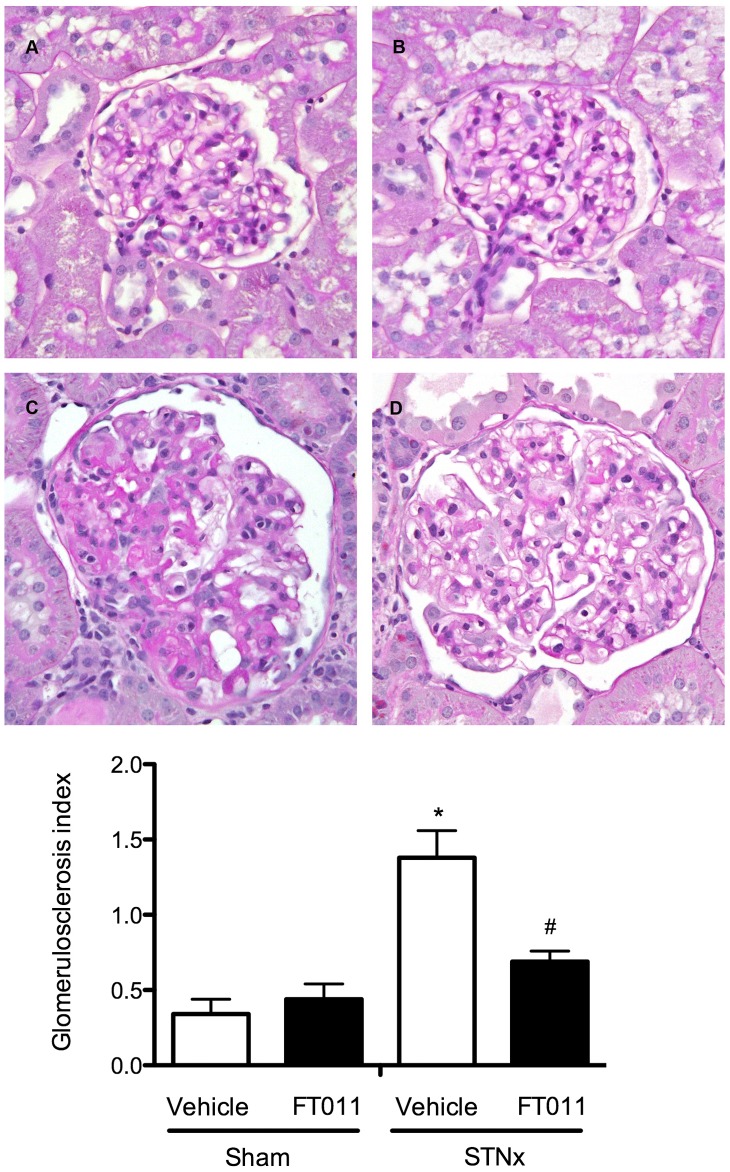
Representative photomicrograph of PAS stained sections from STNx rats. Representative photomicrograph of PAS stained sections from sham and STNx rats treated with and without FT011. In sham (A) and sham treated with FT011 rats (B), there was minimal glomerulosclerosis, while STNx rats (C) was associated with an increase in glomerulosclerosis. Treatment of STNx rats with FT011 (D) was associated with a reduction in extent of glomerulosclerosis. Magnification x400. Glomerulosclerotic index are expressed as mean ± SEM. *P<0.05 compared with sham and sham treated with FT011, **^#^**P<0.05 versus untreated STNx rats.

**Figure 5:**
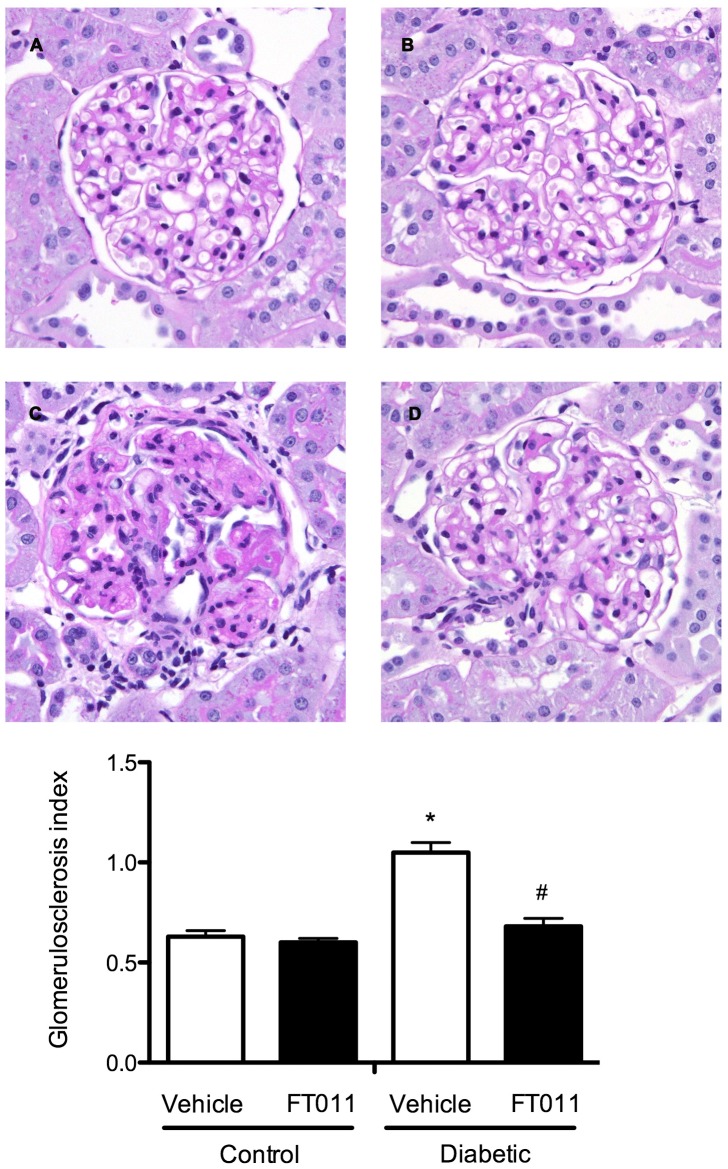
Representative photomicrograph of PAS stained sections from diabetic rats. Representative photomicrographs of PAS stained kidney sections from control (A) and control animals treated with FT011 (B), diabetes (C) and diabetic rats treated with FT011 (D). Compared with control animals, untreated diabetic rats (C) demonstrated marked glomerulosclerosis characterised by diffuse and nodular glomerulosclerosis. FT011 treated diabetic rats (D) demonstrated near normal glomerular histology. Magnification, ×400; Glomerulosclerotic index data are expressed as mean ± SEM. *P<0.05 versus control; **^#^** *P*<0.05 versus vehicle treated diabetic rats.

**Figure 6:**
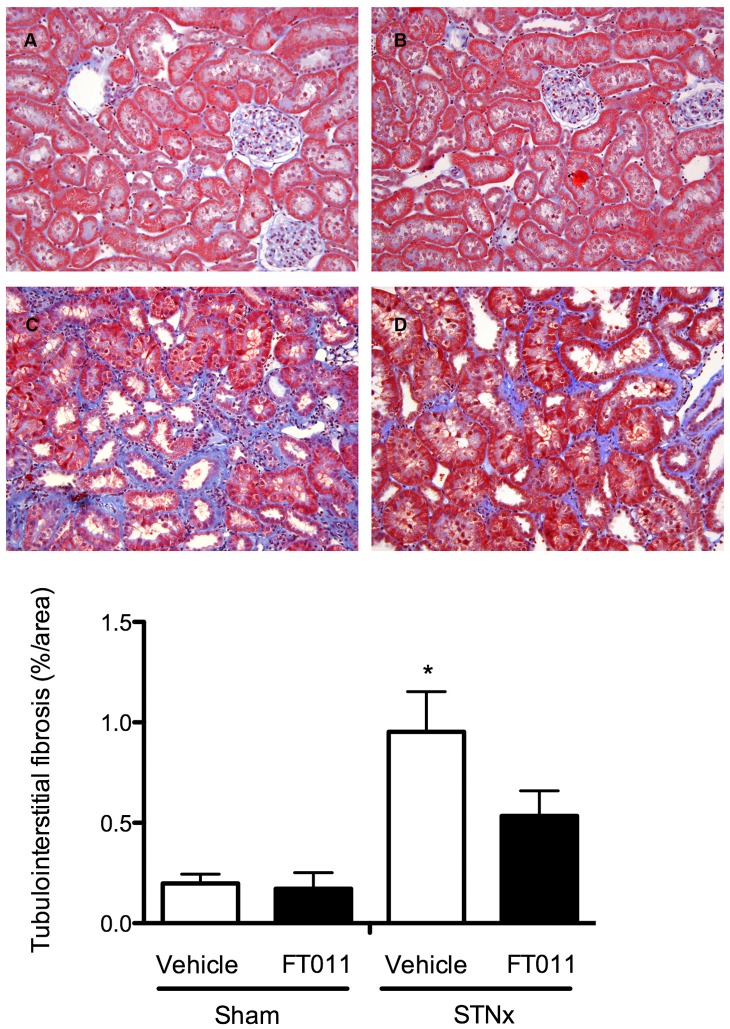
Representative photomicrograph of Masson’s stained sections from STNx rats. Representative photomicrograph of modified Masson’s trichrome-stained sections showing tubulointerstitial fibrosis in sham and STNx rats treated with and without FT011. In sham (A) and sham treated with FT011 (B) there were minimal cortical interstitial fibrosis, while STNx (C) was associated with an increase in interstitial fibrosis (blue). Treatment of STNx rats with FT011 (D) was associated with a trend towards a reduction in tubulointerstitial fibrosis. Magnification x400. Quantitative data are expressed as mean ± SEM. *P<0.05 compared with shams.

**Figure 7:**
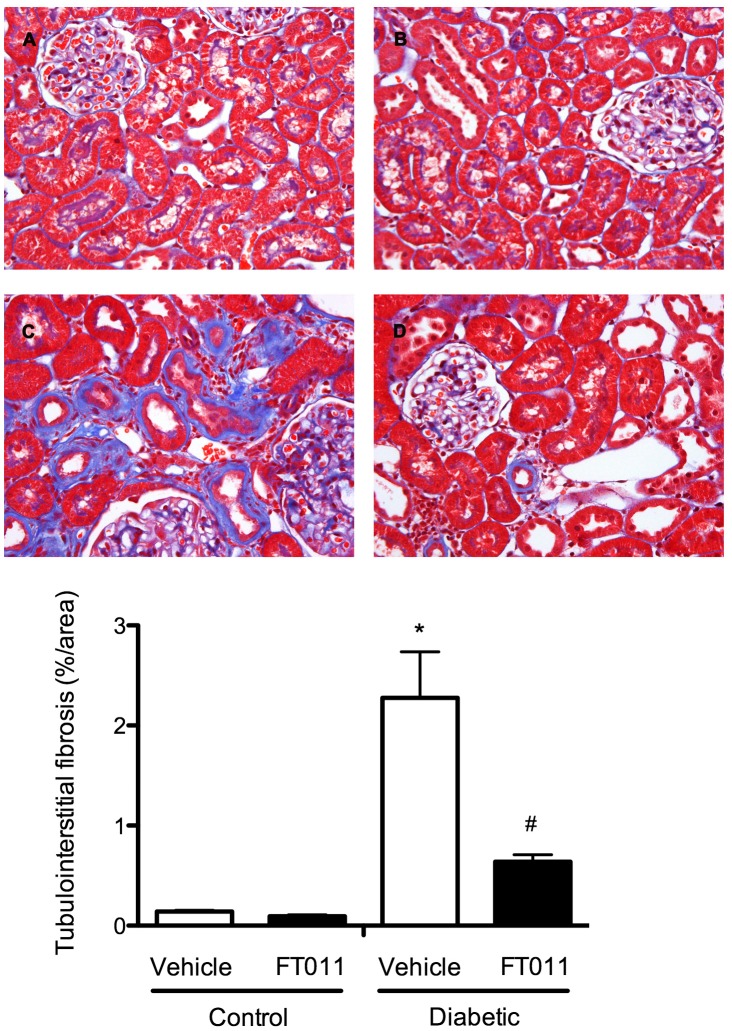
Representative photomicrograph of Masson’s stained sections from diabetic rats. Representative photomicrographs of Masson’s trichrome stained kidney sections from control (A) and control animals treated with FT011 (B), diabetic rats (C) and diabetic rats treated with FT011 (D); compared with control animals, untreated diabetic rats (C) demonstrated severe interstitial fibrosis as blue staining. FT011 treatment in diabetic animals (D) significantly reduced interstitial fibrosis. Magnification, ×400; Quantitative data are expressed as mean ± SEM. *P<0.05 versus control; **^#^** *P*<0.05 versus vehicle treated diabetic rats.

Glomerulosclerosis was a prominent feature in both STNx (Figure 4) and diabetic Ren-2 rats (Figure 5). These changes were attenuated by treatment with FT011 in both disease groups (Figure 4 and Figure 5). Substantial tubulointerstitial fibrosis was also noted in both STNx and diabetic rats (Figure 6 and Figure 7). FT011 reduced tubulointerstitial fibrosis in diabetic ren-2 rats (Figure 7) and trended towards a reduction in STNx rats (p<0.09, Figure 6).

### Interstitial Macrophage Infiltration and Tubular Osteopontin

**Figure 8:**
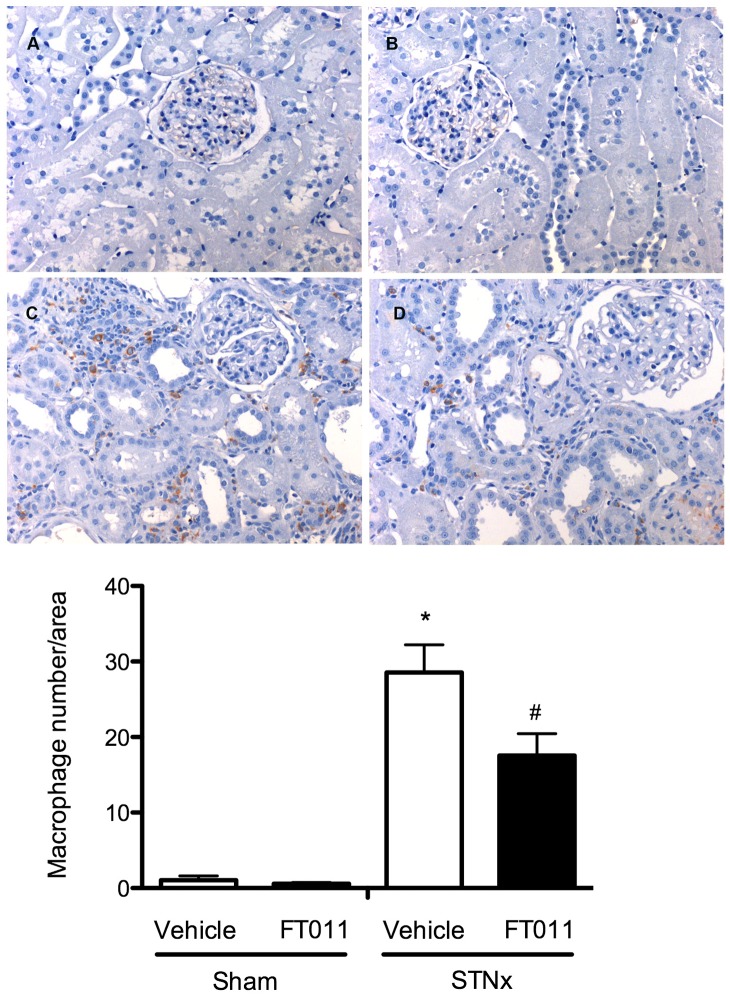
Representative photomicrograph of ED-1 staining from STNx rats. Representative photomicrographs of ED-1 immunostained sections from sham and STNx rats treated with or without FT011. In sham (A) and sham treated with FT011 (B) rats, only occasional macrophages were observed in the interstitium, while STNx rats (C) were associated with numerous macrophages. Treatment of STNx animals with FT011 (D) was associated with a reduction in macrophage number. Magnification x200. Quantitative data are expressed as mean ± SEM. *P<0.05 versus sham; ^#^P<0.05 versus vehicle treated STNx rats.

**Figure 9:**
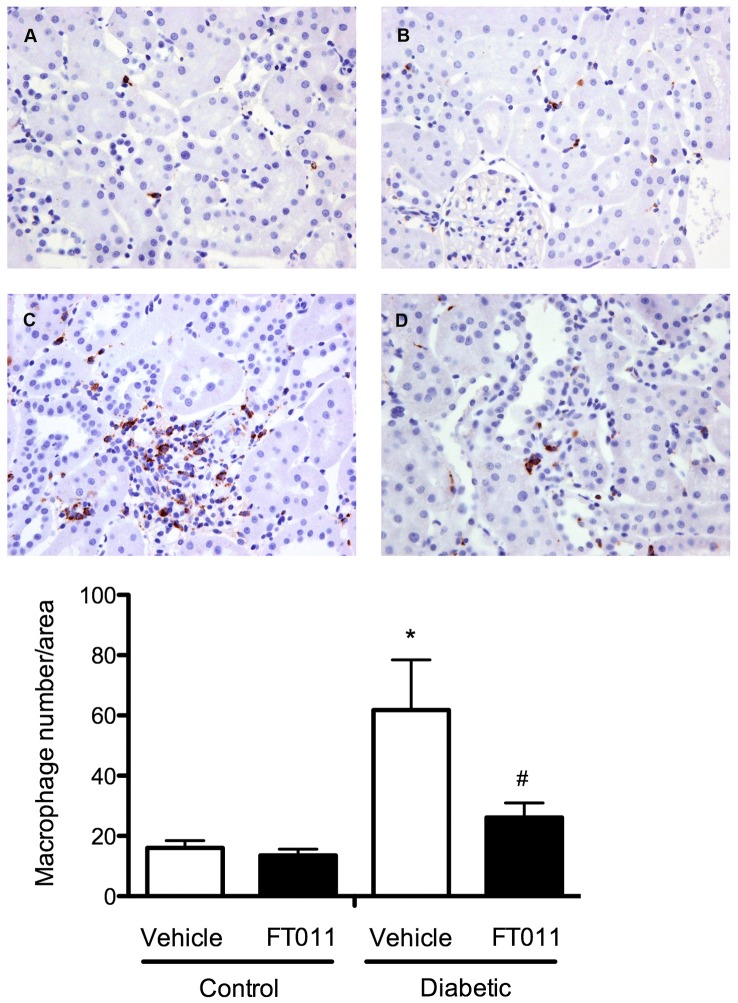
Representative photomicrograph of ED-1 staining from diabetic rats. Representative photomicrographs of ED-1 immunostained sections from control and diabetic rats treated with or without FT011. In control (A) and control treated with FT011 (B) rats, only occasional macrophages were observed in the interstitium, while diabetic rats (C) were associated with numerous macrophages. Treatment of diabetic animals with FT011 (D) was associated with a reduction in macrophage number. Magnification x200. Quantitative data are expressed as mean ± SEM. *P<0.05 versus control; **^#^** *P*<0.05 versus vehicle treated diabetic rats.

**Figure 10:**
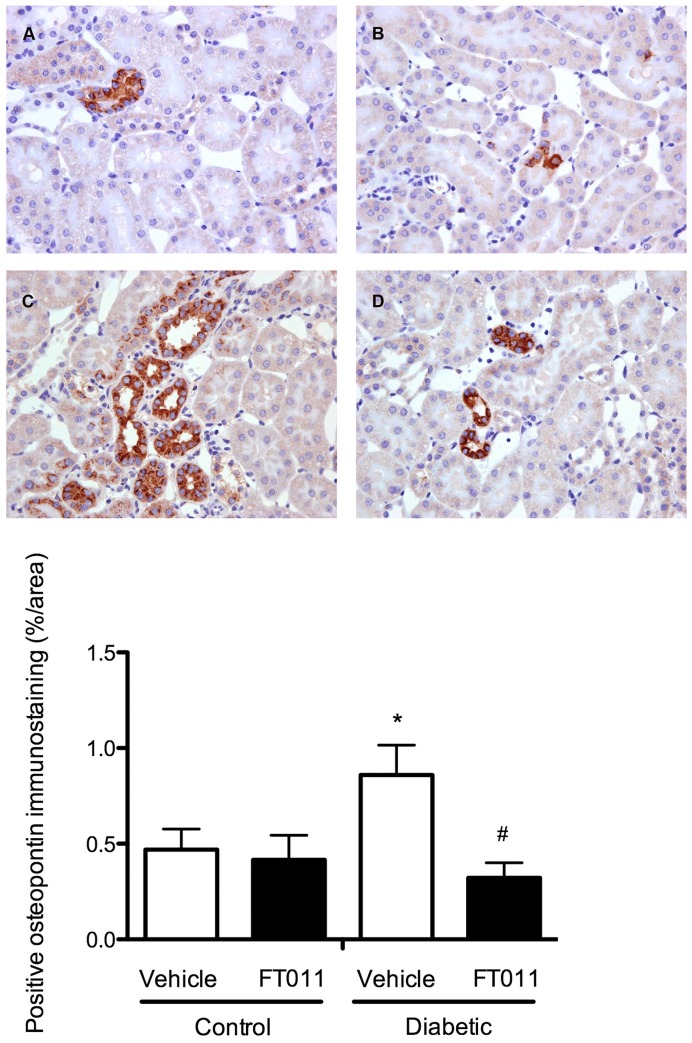
Representative photomicrograph of osteopontin immunostaining from diabetic rats. Representative photomicrographs of osteopontin immunostained sections from control and diabetic rats treated with or without FT011. In control (A) and control treated with FT011 (B) rats, only few tubules were immunostained positively for osteopontin, while diabetic rats (C) were associated with an increased in osteopontin immunostaining. Treatment of diabetic animals with FT011 (D) was associated with a significant reduction in numbers of tubules immunostained positively for osteopontin. Magnification x400. Quantitative data are expressed as mean ± SEM. *P<0.05 versus control; **^#^** *P*<0.05 versus vehicle treated diabetic rats.

Numerous macrophages were detected in the tubulointerstitium in both STNx and diabetic ren-2 rats when compared to control animals (Figure 8 and Figure 9). Treatment of STNx and diabetic ren-2 rats with FT011 was associated with a significant reduction in interstitial macrophage infiltration (Figure 8 and Figure 9). Osteopontin, a macrophage chemoattractant was increased in the tubules of diabetic rats (Figure 10). Treatment of diabetic rats with FT011 was associated with a significant reduction in tubular osteopontin immunostaining (Figure 10).

## Discussion

In addition to inhibiting TGF-ß1 induced collagen production, FT011 was found in the present study to also inhibit the pro-fibrotic and pro-proliferative effects of PDGF-BB. Consistent with these actions, this compound attenuated both the functional and structural manifestations of injury in animal models of non-diabetic and diabetic kidney disease. Like tranilast, the parent compound from which FT011 was derived, the precise mechanism by which FT011 exerts its salutary effects on TGF-ß1 and PDGF-BB activities remain unknown. To the best of our knowledge, FT011 is neither a binding factor nor a receptor antagonist for either PDGF-BB or TGF-ß1. We conjecture that it may modulate intracellular signaling pathways that are either common to or interact with both cytokines, though this remains to be proven.

The remnant kidney (STNx) model of progressive disease shares the major hallmarks of most forms of kidney injury seen in humans, developing hypertension, proteinuria and declining GFR in conjunction with its major histopathological characteristics of glomerulosclerosis, tubulointerstitial fibrosis, tubular atrophy and macrophage infiltration [12]. Moreover, akin to progressive CKD in humans the remnant kidney model also shares its responsiveness to blood pressure reduction [18] and blockade of the RAS [12]. In the present study, we show that FT011 not only attenuated the decline in GFR and reduced proteinuria but also effectively reduced structural injury including a significant reduction in glomerulosclerosis and interstitial macrophage infiltration, and a trend towards reduction in tubulointerstitial fibrosis.

Given the importance of diabetes as the major cause of end stage of renal disease (ESRD) in most industrialised countries, we next examined the effects of FT011 in experimental diabetic nephropathy. These studies were undertaken in the transgenic (mRen-2)27 rat which expresses the mouse renin (Ren-2) gene on a Sprague-Dawley background. While heterozygous Ren-2 rats display only modest changes compared to STNx rats in kidney structure and function, they develop nephropathic changes that are highly reminiscent of human diabetic kidney disease following the induction of streptozotocin (STZ)-diabetes [17]. These include glomerulosclerosis, tubulointerstitial fibrosis and interstitial macrophage infiltration with hypertension and proteinuria. The Ren-2 rat develops glomerular hyperfiltration at 12–16 weeks post STZ, as with early human diabetic kidney disease [18]. As in the remnant kidney model, FT011 markedly attenuated the development of proteinuria as well as reducing fibrosis in both the glomerulus and tubulointerstitium and interstitial macrophage infiltration. Glomerular filtration rate in this model of diabetic nephropathy was unaffected.

Hypertension develops *pari passu* with most forms of CKD and is a consistent feature of STNx rats. Though similar in both treated and untreated groups after 2 weeks of treatment, differences in systolic blood pressure became apparent thereafter. By contrast, FT011 did not lower blood pressure in either hypertensive diabetic or non-diabetic Ren-2 rats nor in normotensive sham- nephrectomised animals. As shown in the study, systolic BP in STNx rats increased by ∼100 mmHg, proteinuria increased to from 21 to 366 mg/day and GFR fell by ∼90%. Given the severity of this injury, the response to FT011 is very significant with proteinuria falling by 180 mg/day, the rise in SBP being reduced by 55 mmHg and GFR improving by more than two-fold. By contrast, diabetic rats had less severe injury with an increase rather than a diminution in GFR, a relatively modest 34 mmHg increase in SBP and an albumin excretion rate of 15 mg/day (∼25 mg of protein/day). In these rats, FT011 reduced SBP by 13 mmHg and did not affect the already elevated GFR. While albuminuria was normalized with FT011, the level in untreated diabetes rats was only one tenth of that found in STNx animals. Together these findings suggest that the lower blood pressure in FT011-treated animals may be a consequence of their better kidney function in STNx rats, rather than a direct hypotensive effect of the drug.

TGF-ß has long been recognised as playing a key role in the pathogenesis of fibrosis for the past 25 years [19], [20] inducing connective tissue formation, not only by stimulating cells already committed to the task of matrix production but also by activating epithelial and endothelial cells to behave similarly [21], [22]. Unsurprisingly, given the importance of fibrosis in the pathogenesis of progressive chronic kidney disease [23], the quest to antagonise TGF-ß came very soon after the descriptions of its biological activity [24]. At present such agents include monoclonal antibodies, anti-sense oligonucleotides, soluble (decoy) receptors and inhibitors of the TGF-ß type I receptor, [25], all of which are in various stages of preclinical and clinical development for a range of fibrotic conditions and malignancies [26].

Despite being able to reduce fibrosis and prevent the decline in GFR, the ability of TGF-ß inhibition to reduce proteinuria, a cardinal manifestation of CKD, is far less certain. For instance, in their proof-of-concept study, Ziyadeh and colleagues showed that a monoclonal anti-TGF-ß antibody prevented mesangial matrix expansion and attenuated the decline in GFR but did not reduce albuminuria in the db/db mouse model of diabetic nephropathy [27]. In a similar study, db/db mice, treated with the TGF-ß type I receptor kinase inhibitor, GW788388, displayed substantial reductions in fibrosis but not proteinuria [28].

In the normal kidney macrophages are mostly restricted to the renal capsule, pelvic wall and the adventitia of large vessels [29]. Macrophage infiltration is, however, a prominent feature in a wide range of kidney diseases where its extent correlates closely with declining renal function [12], [30], [31], [32], [33]. These inflammatory cells elaborate reactive oxygen intermediates, proteases, inflammatory cytokines and growth factors including TGF-β itself [12], [30], [34], all of which may contribute to renal injury [30]. In addition to its well known effects on fibrogenesis, TGF-β also stimulates macrophage chemotaxis by augmenting the chemokine expression. One such TGF-ß inducible chemokine is the glycoprotein, osteopontin [35], [36]. In the present study, osteopontin was abundantly expressed in the proximal tubular epithelial cells in diabetic animals in close proximity to areas of macrophage infiltration, consistent with its actions as a potent chemotactic factor for macrophages [37]. Treatment with FT011 was associated with attenuation in both osteopontin expression and interstitial macrophage infiltration in diabetic animals. While not proving it, such findings are consistent with FT011’s ability to inhibit the biological activities of TGF-β1 and thereby the expression of osteopontin and consequent macrophage infiltration.

In addition to TGF-ß, there are also substantial data implicating PDGF in the progression of CKD. With its four isoforms and two receptors, the PDGF system has been shown to regulate a vast array of pathological events within the kidney, not only stimulating the production of extracellular matrix in a wide range of kidney cells, but also inducing their proliferation [3], [7]. In addition to a range of drugs that include anti-PDGF effects in their spectrum of activity, several specific antagonists of the PDGF system have also been studied in experimental kidney disease that include anti-sense oligonucleotides, neutralising antibodies or oligonucleotide aptamers [7], [38]. These agents have mostly been examined in rats with acute Thy 1.1 nephritis where they have consistently led to a reduction in mesangial cell proliferation and extracellular matrix accumulation [7]. There are no reports, to our knowledge, regarding the effectiveness of PDGF antagonism in either the classical, remnant kidney model of chronic progressive kidney disease or in chronic diabetic nephropathy with the exception of a single report that used the multi receptor tyrosine kinase inihibitor (c-able, c-kit, PDGFR), imatinib, in the apoE knockout mouse with streptozotocin-induced diabetes, wherein proteinuria was reduced [39].

In addition to specific inhibitors, a number of other agents, whose precise mechanisms of action have not been precisely defined, include within their spectra of activity the ability to antagonise both TGF-ß and/or PDGF systems to varying extents. For instance, trapidil, an agent that, among other actions, dose-dependently reduces PDGF-BB induced mesangial cell proliferation, accelerated injury in a model of nephrotoxic nephritis in rabbits [40] and worsened injury in a model of ischemia reperfusion in the rat [41]. Pirfenidone, on the other hand, an agent that inhibits TGF-ß’s and to a lesser extent PDGF’s actions, reduced fibrosis, blood pressure and the rise in serum creatinine in subtotal nephrectomised rats [42]. It did not, however, significantly affect proteinuria in animal or human studies [42], [43], [44], [45].

Akin to pirfenidone, the effects of tranilast have also been examined in both the experimental and human CKD settings. While the precise mechanisms of tranilast’s actions are unknown, this compound has repeatedly been shown to inhibit the actions of both PDGF and TGF-ß [9], [46], [47]. In animal models, the administration of tranilast led to improvements in kidney structure and function, including reductions in proteinuria [48], [49]. Studies in human CKD have been confined to diabetic nephropathy, exploring both early and advanced disease. Among those with early disease (serum creatinine <92 µmol/l (1.2 mg/dl), tranilast, when added to an ACE inhibitor or ARB, significantly reduced albuminuria and urinary collagen type IV excretion, contrasting the placebo group in which both parameters increased over the 12 month study period [50]. In a study of patients in whom serum creatinine >114 µmol/l (1.5 mg/dl), the slope of 1/serum creatinine, a robust marker of change in GFR, was significantly less steep during tranilast treatment than before [51]. Urinary albumin and collagen type IV excretion tended to decrease with time, but did not reach statistical significance in this small 8 patient study.

Given the lack of anti-proteinuric effects with agents that inhibit TGF-ß alone, the findings of the present study in conjunction with those of tranilast, suggest that a broader spectrum of action may be beneficial. Whether PDGF-BB inhibition or other actions of these cinnamoyl anthranilate derivatives account for their combination of anti-proteinuric and other renoprotective effects however, remains uncertain. Notably, however, although FT011 substantially reduced the fibrotic scar in the diseased kidney, no inhibition of wound closure or scar dehiscence was evident following nephrectomy.
